# Gray divorce among migrants and non-migrants in Norway: trends and implications for mental healthcare use

**DOI:** 10.1093/geronb/gbaf118

**Published:** 2025-06-27

**Authors:** Thijs van den Broek, Øystein Kravdal

**Affiliations:** Erasmus School of Health Policy, Erasmus University Rotterdam, Rotterdam, The Netherlands; Centre for Fertility and Health, Norwegian Institute of Public Health, Oslo, Norway

**Keywords:** marital dissolution, register data, longitudinal analysis, mental health, migrants

## Abstract

**Objectives:**

The literature on gray divorce, that is, marital dissolution at age 50 or later, has hitherto had little consideration for differences between migrants and non-migrants. Differing family attitudes and socio-economic characteristics may, however, lead to differences between non-migrants and (specific groups of) migrants in gray divorce risks and in the mental health implications of gray divorce. We assess such differences by comparing native Norwegians and five groups of migrants (from Nordic countries; Western Europe; Eastern Europe; North Africa, Turkey, and Middle East; Asia) in Norway.

**Methods:**

Drawing on administrative data covering Norway’s full population, group-specific age-standardized divorce rates for the period 1990–2018 are calculated. Group-specific trajectories of mental healthcare use around gray divorce are estimated for the shorter period 2008–2018, using linear probability fixed-effects models.

**Results:**

Age-standardized gray divorce rates have risen for native Norwegians. Rates for migrants are somewhat higher, but for migrants from Eastern Europe, from North Africa, Turkey and the Middle East, and from Asia, no increase in gray divorce risks can be noted. Mental healthcare use rises in the years prior to divorce, peaks in the year of divorce and then declines again. This pattern is largely similar for native Norwegians and the five migrant groups.

**Discussion:**

Despite typically more negative attitudes toward divorce in several migrant communities, gray divorce rates tend to be higher in some migrant groups than among native Norwegians. The mental healthcare use trajectories around gray divorce in native Norwegians and in the migrant groups considered are highly similar.

Gray divorce is typically defined as a marital dissolution at age 50 or later. In the United States (Brown & Lin, 2012, 2022) and Europe (Bildtgård & Öberg, 2025; Dörfler-Bolt et al., 2022; Prioux & Barbieri, 2012; Van den Broek, 2024), divorce rates among people aged 50+ have increased notably since the 1990s. This demographic trend led to rising scholarly attention for the implications of later-life marital dissolutions, including mental health trajectories around gray divorce. This work tends to show that mental health declines in the years before gray divorce, but then often improves again in the years after the marital dissolution (Hu et al., 2024; Lin et al., 2019; Tosi & Van den Broek, 2020).

Work on trends in gray divorce and on mental health trajectories around gray divorce has hitherto mostly focused on the general populations aged 50+ of the United States and various European countries, with no distinction being made between people with and without a migration background. However, as further elaborated upon later, differences in family attitudes and socio-economic resources may be expected to lead to differences between non-migrants and (specific groups of) migrants in both the rates of later-life marital dissolution and the mental health implications of gray divorce. Such differences are relevant, as they may reinforce the mental health disadvantage of older migrants relative to their non-migrant counterparts in high-income countries (Marin et al., 2022).

Norway provides a valuable context for addressing this gap in the scholarly literature, as the country has a rapidly growing population of older migrants, and there are also high-quality administrative data that enable population-wide analyses across both migrant and non-migrant groups. In this study, we (a) compare trends in the gray divorce risks for native Norwegians with the corresponding trends for migrants in Norway from five country clusters (Nordic countries; Western Europe; Eastern Europe; North Africa, Turkey, and Middle East; Asia), and (b) assess whether the trajectories of mental healthcare use around gray divorce differ between native Norwegians and older migrants.

## Background

### Older migrants in Norway

Over the past decades, Norway’s population, including the older subpopulation, has become increasingly ethnically diverse (Dzamarija, 2022). The largest groups of foreign-born older adults are people originating from Denmark, Sweden, and Poland (Dzamarija, 2022). Largely due to labor migration from the late 1960s onwards and subsequent family reunifications, there are also considerable groups of older migrants from countries like Turkey, Morocco, and Pakistan (Martens, 2018).

Migrants from the latter countries were socialized in societies where, compared to Norway, family norms were generally much more conservative, and stances toward divorce were more negative (Engin et al., 2020; Inglehart, 2006). To a lesser extent, this also applies to older migrants from Eastern European countries, such as Poland or Romania (Fučík, 2020; Inglehart, 2006). In contrast, acceptance of divorce is relatively high in the Nordic countries and Western Europe (Fučík, 2020; Kalmijn & Uunk, 2007).

Socio-economic characteristics and living conditions also differ notably across older migrant groups. Older migrants from origins other than the Nordic countries or Western Europe have relatively low incomes, levels of education, and rates of homeownership (Dzamarija, 2022). As argued in the next subsections, there are reasons to suspect that these attitudinal and socio-economic differences may result in variations between non-migrants and certain migrant groups in both (trends in) gray divorce rates and mental healthcare use trajectories around gray divorce.

### Rising gray divorce rates

Between 1990 and 2010, the one-year divorce risk for married people aged 50+ in the United States doubled from approximately 5 per 1,000 to approximately 10 per 1,000 (Brown & Lin, 2012), after which it stabilized at this high level (Brown & Lin, 2022). Brown and Lin (2012) labeled this demographic trend, which they linked to developments such as the growing economic independence of women and the increased acceptance of divorce, as the *gray divorce revolution*. In European countries such as France (Prioux & Barbieri, 2012), Austria (Dörfler-Bolt et al., 2022), Sweden (Bildtgård & Öberg, 2025), and the Netherlands (Van den Broek, 2024) gray divorce rates have also increased.

The differences described above between some groups of older migrants in Norway and their native Norwegian counterparts in attitudes toward divorce and in socio-economic resources may be expected to create differences in gray divorce rates. Barriers to later-life divorce may be lower in groups where divorce is more commonly perceived as an acceptable solution to an unpleasant marriage (Fučík, 2020; Uhlenberg & Myers, 1981). This acceptability may partly involve a snowball effect where a high prevalence of marital dissolution among friends or siblings may further normalize divorce (Büyükkeçeci & Leopold, 2021; De Vuijst et al., 2017; McDermott et al., 2013).

Customs and norms in the country of origin may also shape older migrants’ predisposition toward divorce in their destination countries. Furtado et al. (2013) showed that divorce risks in the United States were relatively low for migrants from countries with low divorce rates, which they interpreted as evidence that cultural factors shape migrants’ likelihood of divorcing. Assuming that this mechanism also applies to middle-aged and older migrants, it may be expected that, compared to their native Norwegian counterparts, older migrants originating from country clusters where divorce is less accepted (e.g., North Africa, Turkey, and the Middle East; Eastern Europe; Asia (cf Engin et al., 2020; Fučík, 2020; Inglehart, 2006; Kalmijn & Uunk, 2007)) may have higher (increases since the 1990s in) age-standardized divorce rates, whereas this may not be the case for older migrants from country clusters where divorce is more accepted (e.g., Nordic countries; Western Europe (cf Fučík, 2020; Inglehart, 2006; Kalmijn & Uunk, 2007)).

On the other hand, lower rates of premarital cohabitation in many migrant groups may contribute to raised gray divorce risks, because, in groups where cohabitation is a commonly accepted option, those who do marry may have particularly conservative family values or high-quality relationships, which likely reduces the probability of a break-up (cf Kulu & Boyle, 2010). Migration-related stressors may furthermore contribute to marital strain (cf Andersson et al., 2015). The lower levels of education, income, and home-ownership in many groups of non-Western older migrants (Dzamarija, 2022) may also be reflected in higher gray divorce rates, as these factors are known antecedents of gray divorce (Alderotti et al., 2022; Lin et al., 2018).

### Gray divorce and mental health

Recent longitudinal empirical work showed that gray divorce tends to have detrimental mental health implications (Lin et al., 2019; Tosi & Van den Broek, 2020), which may lead to a higher uptake of mental healthcare (Krzyż et al., 2023). Amato (2000) argued that the implications of divorce are best understood from a divorce-stress-adjustment perspective within which two models are embedded: the crisis model and the chronic strain model. Central to the divorce-stress-adjustment perspective is the perception of divorce not as a discrete event, but rather as a process that already starts in the years prior to the formal marital dissolution and that may end long after the formal end of the marriage.

The crisis model posits that divorce can be a substantial source of stress, for example, due to heightened emotional distress or financial challenges (Amato, 2000). The model recognizes that stress may not just be accumulated around the time of divorce, but also in the period preceding it (Booth & Amato, 1991). Marriages often are already strained in this period, which may harm mental health (Goldfarb & Trudel, 2019). Accordingly, longitudinal studies have reported raised depressive symptoms (Tosi & Van den Broek, 2020), loneliness (Kapelle & Monden, 2024), and antidepressants use (Hu et al., 2024) already in the years prior to gray divorce (cf Kravdal et al., 2023).

In the crisis model, the buildup of stress in the periods preceding and around the time of divorce is followed by adjustment. Divorced persons may adapt to their new status by developing new routines, coping strategies, and social support networks (Amato, 2000). Re-partnering can benefit the adjustment process. It is associated with improvements in life satisfaction (Gloor et al., 2021) and, at least for women, may mitigate the negative economic consequences of divorce (Hogendoorn & Kalmijn, 2024; Lin & Brown, 2021). Successful post-divorce adjustment is likely to be reflected in mental health recovery. Accordingly, longitudinal studies in the United States (Lin et al., 2019) and the UK (Tosi & Van den Broek, 2020) have provided evidence of recovery after gray divorce with regard to depressive symptoms.

Although the divorce-stress-adjustment perspective was not developed specifically to study the implications of later-life marital dissolutions, it considers that adjustment to divorce may be particularly challenging for older persons, for example, due to their stronger emotional and financial investments in their marriages and bleaker prospects of remarriage (Wang & Amato, 2000). In line with this idea, Lin et al. (2019) emphasize that mental health recovery after gray divorce tends to be protracted, a pattern which they refer to as *convalescence* (cf Lin & Brown, 2020). Complete mental health recovery may also not be achieved at all. In contrast to the crisis model and the convalescence model, the chronic strain model accordingly postulates *persistent* stress following divorce, for example, due to lasting economic hardship or loneliness (Amato, 2000). Consistent with this notion, Hu et al. (2024) showed that although older adults’ use of antidepressants in Finland declined from around the time of divorce, it did not return to the substantially lower levels observed a few years pre-divorce. Similarly, Kapelle and Monden (2024) found that, in Australia, loneliness levels peaked in the year of gray divorce, then declined in subsequent years, but remained slightly elevated.

Differences in value orientations between native Norwegians and various groups of older migrants may be expected to be reflected in heterogeneity in the mental health impact of gray divorce. When one’s behavior is discrepant with one’s values about divorce, then this may yield the inherently uncomfortable experience of cognitive dissonance (Festinger, 1957). Accordingly, adjustment to divorce may be more difficult for people who held less favorable attitudes toward marital dissolution prior to divorce (Wang & Amato, 2000). The stigmatization hypothesis proposed by Kalmijn and Uunk (2007) moreover suggests that individuals who violate common norms about divorce may face negative social responses, for example, labeling, isolation and status loss (cf Aquino et al., 2022). Experiences of stigmatization are, in turn, likely to be detrimental for one’s mental health.

Furthermore, the lower incomes and levels of education in many groups of non-Western older migrants (Dzamarija, 2022) should be considered. Individuals with low incomes and low levels of education may be pushed into a precarious economic situation if they no longer can share the scale advantages associated with partnership (cf Hogendoorn et al., 2020). This may compromise their mental health (Grundy et al., 2019). Hence, the implications of gray divorce for mental healthcare use may be most pronounced in clusters of older migrants with typically relatively poor socio-economic resources and originating from regions where divorce is generally viewed relatively negatively (e.g., North Africa, Turkey, and the Middle East; Eastern Europe; Asia).

### Data

We draw on administrative data from multiple sources covering the full population of Norway. The register extractions available for our analysis include the period up to January 1st, 2019. Data for the period 1990–2018 are used to assess trends in gray divorce risks in various origin groups. Information on marital status, year of birth, sex, country of birth, and year of death is taken from the Norwegian Population Register, which includes all persons who have ever lived in Norway after 1964.

For a second set of analyses, we use information on mental healthcare use derived from the Control and Distribution of Health Reimbursement database (KUHR) and the Norwegian Patient Register (NPR), and link this with information from the Population Register via the unique personal identification number. Following prior work (e.g., Kravdal et al., 2024), we coded individuals as mental healthcare users in a particular year when the KUHR or NPR registers indicated that in the specific year they had had at least one face-to-face general practice consultation where a symptom or diagnosis of a mental health problem was reported (International Classification of Primary Care (Version 2, ICPC-2) codes P01-P99), or at least one specialist consultation where a mental disorder (International Classification of Disease (version 10) codes F01-F99) was reported as a main or secondary diagnosis. Given that complete information on mental healthcare consultations is not available for the years prior to 2008, our analyses of the mental health implications of gray divorce draw on data covering the period 2008–2018.

## Methods

Our first set of analyses is largely descriptive. It sheds light on group-specific trends in the age-adjusted divorce rate among people aged 50+, whereby we distinguish Norwegian-born people aged 50+ and their foreign-born counterparts from five country clusters (Nordic countries; Western Europe; Eastern Europe; North Africa, Turkey, and Middle East; and Asia). An overview of the origin countries per cluster is provided in Supplementary Table 1. Note that people born in the Americas, Oceania, or Sub-Saharan Africa were not considered in our study, due to small numbers of married people aged 50+ from these groups in the Norwegian population. The second set of analyses aims to assess the mental healthcare use implications of gray divorce. The same six origin groups are distinguished.

### Description of trends

We estimate age-standardized one-year marital dissolution probabilities for women and men in different origin groups in different periods, using the age distribution of Norwegian-born married women and men in the 2000–2004 period as the standard. For this purpose, we first produce a series of one-year observations for women and men from the beginning of 1990 to the end of 2018, with the following additional restrictions: (a) turning 50–79 within the year, (b) being resident in Norway at the start of the year, (c) being still alive at the end of the year, (d) being married (but not formally separated) at the start of the year, and (e) coming from one of the country groups that we consider. Meeting these criteria were 24,926,699 person–year observations nested in 1,980,937 persons.

Based on these observations, group- and period-specific age-standardized one-year marital dissolution probabilities are calculated as follows:


(1) $$D_{\mathrm{sgy}}=\sum_{a=50,\;75}(p_{sa}*d_{\mathrm{sgya}})$$

In Equation (1), $d_{\mathrm{sgya}}$ is the probability of becoming formally separated or divorced within the calendar year for individuals of sex *s* in origin group *g*, in period *y* (1990–1994, 1995–1999, 2000–2004, 2005–2009, 2010–2014, or 2015–2018) and in age group *a* (50–54, 55–59, 60–64, 65–69, 70–74, and 75–79). Here, formal separation refers to the legal dissolution of a marital union short of divorce, as recognized by Norwegian law. This is a common and typically legally required first step toward formal divorce in Norway, as the law mandates a one-year period of formal separation before a divorce can be granted, although certain exceptions to this rule may apply. The weights $p_{sa}$ are the proportion of the one-year observations in age group *a* for Norwegian-born people of sex *s* in the years 2000–2004.

### Modeling mental health implications

The analytical sample for our analyses of mental healthcare use is restricted to individuals who were married and aged 50+ at some point between 2000 and 2018, and still alive until at least 2009. For individuals meeting these criteria, person–year observations were coded for each year between 2008 and 2018 in which they were alive and resided in Norway throughout the year. For individuals who became widowed, we dropped the observation for the year of death of the spouse and subsequent person–years. If people had a second marital dissolution in the observed period, the person–year observations from the year of the second divorce onwards were dropped as well. Finally, persons with only a single person–year observation meeting these inclusion criteria were excluded. This procedure resulted in a final analytical sample of 10,334,997 person–year observations nested in 1,216,531 persons, of whom 87,014 went through divorce during the observation period. Descriptive statistics by origin group and sex are presented in Table 1.

**Table 1.** Descriptive statistics.

Variable	Norway	Other Nordic countries	Rest of Western Europe	Eastern Europe	North Africa, Turkey, and Middle East	Asia (except Middle East)
*Women*						
**Mental healthcare use**	12.98%	13.13%	13.25%	16.67%	20.56%	11.43%
**Age in years, Mean (SD)**	62.81	62.31	61.98	57.95	57.46	57.77
	(7.95)	(8.06)	(8.12)	(6.59)	(6.42)	(6.38)
**Number of person–year observations**	4,631,774	84,627	59,326	78,614	33,414	95,830
**Number of persons (Of whom persons going through divorce)**	541,949	10,539	7,488	10,858	4,792	12,944
	(31,652)	(783)	(567)	(1,126)	(549)	(1,031)
*Men*						
**Mental healthcare use**	8.35%	9.97%	9.40%	10.49%	19.15%	10.77%
**Age in years, Mean (SD)**	63.49	61.86	61.54	57.84	57.94	59.48
	(8.07)	(7.76)	(7.75)	(6.70)	(6.77)	(7.22)
**Number of person–year observations**	4,938,242	79,998	79,670	98,072	63,212	92,218
**Number of persons (Of whom persons going through divorce)**	573,382	10,088	10,011	13,924	8,954	11,602
	(46,060)	(1,144)	(1,056)	(788)	(1,280)	(978)

*Note*. SD = standard deviation.

When estimating mental health effects of divorce, it must be taken into account that many factors of importance for the divorce probability may also have an impact on mental health (Amato, 2000). Unfortunately, many of these confounders are unobserved, but we control for unobserved factors that are constant over time by estimating fixed-effects linear probability models (Allison, 2009). The models are specified as follows:


(2) $$Y_{it}=\beta_{k}D_{it}^{\left(k\right)}+\;\gamma A_{it}+v_{i}+\varepsilon_{it}$$

The outcome $Y_{it}\;$ is 1 if person *i* had at least one mental healthcare consultation in year *t*, otherwise 0. The main explanatory variable *D* is a categorical variable indicating time since formal separation or, in the case of direct divorce, since divorce. The *k* categories considered are 5+ years pre-divorce (reference), 3–4* *years pre-divorce, 1–2* *years pre-divorce, year of divorce, 1–2* *years post-divorce, 3–4* *years post-divorce, and 5+ years post-divorce. The fixed effects $v_{i}$ account for all time-invariant characteristics of individuals, regardless of whether these characteristics are observed (Allison, 2009). With this type of model, we essentially regress the within-person variation in the outcome on the within-person variation in *D*.

We include $A_{it}$, a categorical variable for age with 31 one-year categories from age 50–80, to capture age-related changes in mental healthcare use. This is important, because as time since divorce increases, age also increases, and both may affect mental health. If we only included individuals who experienced divorce, this would create a linear dependence problem: current age minus time since divorce equals age at divorce, which is constant for each individual and therefore absorbed by $v_{i}$ (Kravdal et al., 2023). The age effect would be estimated from the variation in mental health within the categories of two (1–2 and 3–4) or more years (5+) for the time-since-divorce variable, while that variation may largely be a result of increasing time since divorce. By also including individuals who remain continuously married throughout the observation period, we avoid this problem. These stably married individuals remain in the reference category for the time-since-divorce variable and therefore do not contribute directly to the estimate of its effect. However, their mental healthcare use across ages helps identify the age effect more reliably. This approach allows us to better isolate the specific impact of time since divorce from general age-related trends in mental healthcare use.

All models are estimated with cluster robust standard errors to account for our data’s nested nature. Given that the consequences of gray divorce may differ between women and men (e.g., Büyükkeçeci & Leopold, 2024; Lin & Brown, 2021), models are estimated separately not only by origin group, but also by gender. Additionally, we estimate gender stratified fixed-effects models in which all origin groups are pooled and every term is interacted with origin group dummies to determine whether the group differences in the coefficient estimates are statistically significant (cf Van den Broek & Fleischmann, 2022).

## Results

### Age-standardized gray divorce rates

**Figure 1.**
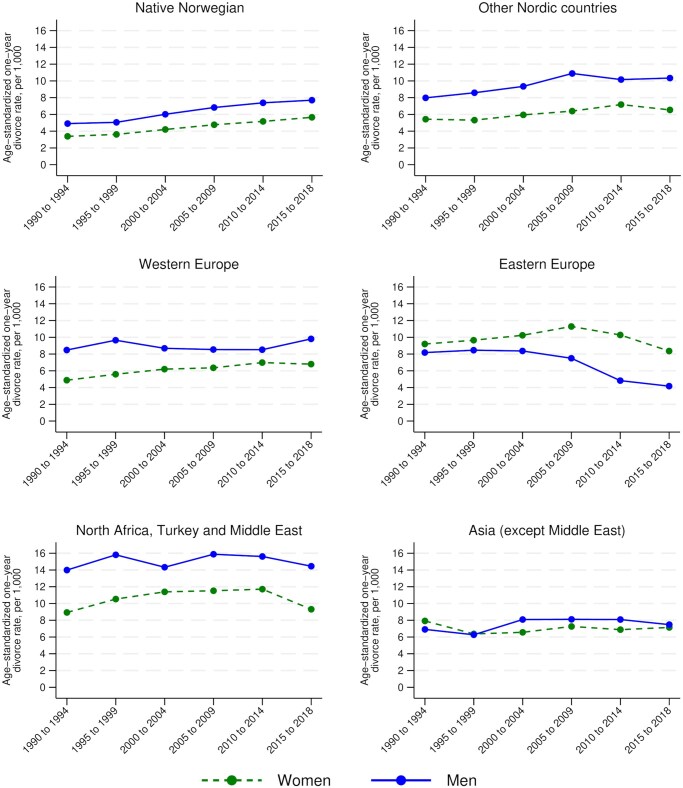
Trends in age-standardized one-year divorce rates by sex and origin group.

Figure 1 shows how divorce risks for married native Norwegians aged 50+ and their counterparts in migrant groups have developed since the 1990s. For native Norwegians, the age-standardized one-year divorce rates increased from 3.4 per 1,000 women and 4.9 per 1,000 men in the period 1990–1994 to 5.7 per 1,000 women and 7.7 per 1,000 men in the period 2015–2018. Although substantial, this rise is notably smaller than the aforementioned increase in gray divorce rates observed by Brown and Lin (2012, 2022) for the United States over the same period. In contrast to our expectations, age-standardized divorce rates were higher in all migrant groups compared to native Norwegians. The divorce risks were particularly high for people from North Africa, Turkey, and the Middle East.

For almost all groups, the age-standardized gray divorce risks were higher for men than for women. This is due to the declining divorce risks with higher ages in combination with the fact that in heterosexual marriages, the male spouse tends to be older than the female spouse. An exception is the pattern for migrants from Eastern Europe, where women’s age-standardized divorce rates exceeded men’s. Plausibly, this reflects that marriages with a native Norwegian have been more common for Eastern European women than for men (Flemmen, 2008), and divorce risks are known to be elevated in mixed marriages (Andersson et al., 2015; Dupont et al., 2020). We therefore also calculated age-standardized divorce rates only for individuals in homogamous marriages, that is, with a spouse from the same origin group (see Supplementary Figure 1). The estimated divorce risks for women from Eastern Europe were lower than the corresponding rates for their male counterparts when focusing on homogamous couples only, as in the other origin groups. Compared to the results presented in Figure 1, the age-standardized divorce rates in homogamous couples tend to be lower. For most migrant groups, these rates nevertheless still clearly exceed the age-standardized divorce rates observed in native Norwegian people.

### Fixed-effects regression analyses

**Figure 2.**
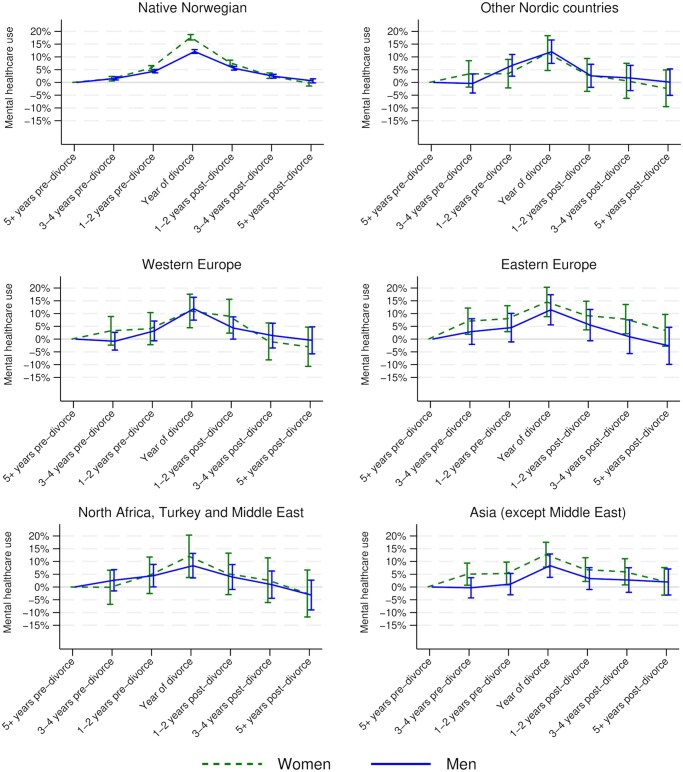
Estimated age-adjusted trajectories of mental healthcare use around gray divorce by sex and origin group, with 95% confidence intervals.

The main results of the fixed-effects analyses of mental healthcare use are presented in Figure 2 (for full results, see Supplementary Table 2 (for women) and Supplementary Table 3 (for men). For native Norwegians, the analyses show that, relative to the period of 5+ years prior to divorce, the likelihood of mental healthcare use already rises slightly, but statistically significantly, 3–4* *years prior to divorce and, more substantially, 1–2* *years prior to divorce. Mental healthcare use then peaks in the year of divorce, with this increase being even larger for women than for men. Subsequently, a gradual recovery can be noted for both women and men, and eventually, in the 5+ years post-divorce category, the estimated change in mental healthcare use relative to the baseline level is indistinguishable from zero, and this estimate is highly precise. Hence, there seems to be no substantial systematic persistent mental healthcare use implications of gray divorce for native Norwegians.

Due to the smaller sample sizes, the estimates of the mental healthcare use trajectories around gray divorce for the five migrant groups are less precise. Nevertheless, the pattern of peaking likelihoods of mental healthcare use in the year of divorce and subsequent recovery can be noted for both women and men across origin groups. Post-estimation indicated that the peak in mental healthcare use in the year of divorce relative to the 5+ years pre-divorce baseline period was statistically significantly smaller for women born in Western Europe (Δb: −0.066, 95% CI: −0.133 to −0.000, *p *<* *.05) or Asia (Δb: −0.050, 95% CI: −0.100 to −0.001, *p *<* *.05) than for native Norwegian women. The estimates of the peak increase in the year of divorce relative to the 5+ years pre-divorce baseline period for women and men in all other migrant origin groups were also smaller than the corresponding estimates for their native Norwegian counterparts, but none of these differences were statistically significant. The analyses thus do not provide any evidence in support of our expectation that gray divorce is particularly detrimental for the mental health of older migrants from Eastern Europe, from North Africa, Turkey, and the Middle East, and from Asia.

### Robustness checks

Several additional analyses were performed to assess the robustness of our main results. First, all analyses were repeated with a more restrictive alternative outcome in which a general practice consultation related only to a symptom of a mental health problem, and not a diagnosis, was not coded as mental healthcare use (see Supplementary Figure 2). Given that fertility differs across the groups considered here (Tønnessen, 2020) and that the mental health effects of gray divorce may be more pronounced for parents than for childless persons (Tosi & Van den Broek, 2020), we furthermore estimated models on a sample restricted to (person–year observations in which individuals were) parents (see Supplementary Figure 3). The results of both sets of additional analyses were very similar to the results presented in Figure 2, underscoring the robustness of our main findings.

In the main analyses, we did not consider whether individuals remarried after divorce, because, in our view, re-partnering and new marriages are potential elements of adjustment to divorce. Nevertheless, we also re-estimated all models with a more restrictive analytical sample censored on the year of a new marriage to assess whether post-divorce mental health recovery can also be observed net of new marriages. In support of this idea, the results of the additional analyses (see Supplementary Figure 4) were again almost identical to the results presented in Figure 2.

## Discussion

The gray divorce literature has paid little attention to differences between migrants and non-migrants. Differing family attitudes and socio-economic characteristics may, however, be expected to lead to differences between non-migrants and (specific groups of) migrants in gray divorce risks and in the mental health implications of such divorces. Drawing on administrative data, we therefore assessed whether such differences could be observed between native Norwegians and five groups of migrants in Norway.

When focusing on the native Norwegian majority among married people aged 50+, a gradual increase in age-standardized gray divorce rates could be noted from the 1990s to the 2010s. This is consistent with findings for other high-income countries (Brown & Lin, 2022; Dörfler-Bolt et al., 2022; Prioux & Barbieri, 2012; Van den Broek, 2024). The relative increase over the observed period and the level that the divorce rate reached by the end of this period were, however, both substantially lower than the corresponding estimates reported by Brown and Lin (2022) for the United States. Hence, the gray divorce revolution appears to have been taking place at a considerably slower pace in Norway than in the United States.

Our results showed that gray divorce risks were relatively high for people from North Africa, Turkey, and the Middle East, despite their presumably quite negative attitudes toward divorce. This possibly reflects that migration-related stressors may contribute to marital strain in this group (cf Andersson et al., 2015) and that other risk factors for divorce, for example, having a financial strain and not owning one’s home (Alderotti et al., 2022; Lin et al., 2018) are also relatively common (Dzamarija, 2022).

Across all groups considered, our analyses of mental healthcare use suggest that gray divorce and the processes leading to it trigger a deterioration of mental health, followed by a protracted recovery process. This pattern is in accordance with Lin et al.’s (2019) notion of convalescence (cf Lin & Brown, 2020). The mental healthcare use trajectories around gray divorce for women and men in the five migrant groups largely resembled the patterns for native Norwegians, although estimates were less precise due to the smaller sample sizes. The increase in mental healthcare use in the year of divorce, compared to 5+ years pre-divorce, was significantly smaller for women born in Western Europe or in Asia than for native Norwegian women. For women and men in other migrant groups, this increase was also smaller than for native Norwegians, albeit not statistically significantly so. We thus found no support for our hypothesis that the mental healthcare use implications of gray divorce are most pronounced for clusters of older migrants with typically less socio-economic resources and originating from regions where divorce is generally viewed relatively negatively (e.g., North Africa, Turkey, and the Middle East; Eastern Europe; Asia). It should be noted that marriage rates in the Nordic countries and Western Europe are relatively low (Kalmijn, 2007). Plausibly, individuals from these regions who do choose to marry are especially committed to marriage. This could make divorce particularly stressful for them and mitigate group differences in the mental healthcare use implications of gray divorce.

A key strength of our study is the use of administrative data covering Norway’s entire population. These data enabled us to perform quite detailed analyses of the mental healthcare use trajectories around gray divorce in five subgroups of migrants, which would have been unfeasible with survey data. There are, however, also data limitations. First, the outcome of our fixed-effects analyses is mental healthcare use. Mental healthcare use reflects a combination of having a mental health problem (which has been the focus of prior survey-based investigations of the effects of gray divorce (Lin et al., 2019; Tosi & Van den Broek, 2020) and seeking professional help for this problem. Our fixed-effects modeling strategy accounts for the extent that immigrants are generally less inclined to seek professional help than natives, for example, because of stigma surrounding mental health issues or language barriers (Abebe et al., 2017). However, it is possible that the apparently weaker effect of divorce on mental health care use among immigrants than among natives partly reflects that the distress around the divorce increases, especially the immigrants’ barriers to seeking help for their problems.

There may furthermore be some heterogeneity within the origin country clusters we have chosen, which have also been used in prior migrant health research (Laue et al., 2023). For example, migration motives, family norms, and socioeconomic resources may differ between immigrants from various countries within a cluster. Unfortunately, it was impossible to analyze separate countries because of the modest numbers of gray divorcees, but the strong similarity in the estimates across the groups indicates that it is unlikely that slight changes in the definitions of the country clusters would substantially alter our results. Future research adopting a qualitative approach may shed additional light on the variations in the effects of gray divorce between immigrants from various specific countries.

Although our results suggest that, across the groups considered, the mental health of people recovers in the years after divorce, our data do not enable us to assess the mechanisms involved in the adjustment process. Relevant factors that could not be taken into account here may include the establishment of new social networks (Amato, 2000) or successful strategies for the recovery of financial well-being (Hogendoorn & Kalmijn, 2024). Survey-based longitudinal research or qualitative work is needed to identify and disentangle these mechanisms.

Despite these limitations, our results underscore the importance of cultural and demographic contexts in shaping gray divorce trends and mental health outcomes. While native Norwegians and migrants show largely similar recovery trajectories, the high divorce rates in several migrant groups highlight the need for tailored interventions addressing their specific challenges.

## Supplementary Material

gbaf118_Supplementary_Data

## Data Availability

The data for this study encompass the Norwegian Population Register, the Norwegian Control and Payment of Health Reimbursements Database, and the Norwegian Patient Register. The authors cannot share these data with other researchers due to their sensitive nature and potential for identification. Researchers can access the data by application to the Regional Committees for Medical and Health Research Ethics and the data owners (Statistics Norway and Norwegian Health Directory). The studies reported here were not preregistered.

## References

[gbaf118-B1] Abebe D. S. , Lien L., Elstad J. I. (2017). Immigrants’ utilization of specialist mental healthcare according to age, country of origin, and migration history: a nation-wide register study in Norway. Social Psychiatry and Psychiatric Epidemiology, 52, 679–687. 10.1007/s00127-017-1381-1 28378064

[gbaf118-B2] Alderotti G. , Tomassini C., Vignoli D. (2022). ‘Silver splits’ in Europe: The role of grandchildren and other correlates. Demographic Research, 46, 619–652. 10.4054/DemRes.2022.46.21

[gbaf118-B3] Allison P. D. (2009). Fixed effects regression models. Sage.

[gbaf118-B4] Amato P. R. (2000). The consequences of divorce for adults and children. Journal of Marriage and Family, 62, 1269–1287. 10.1111/j.1741-3737.2000.01269.x

[gbaf118-B5] Andersson G. , Scott K., Obućina O. (2015). Marriage and divorce of immigrants and descendants of immigrants in Sweden. Demographic Research, S18, 31–64. 10.4054/DemRes.2015.33.2

[gbaf118-B6] Aquino T. , Brand J. E., Torche F. (2022). Unequal effects of disruptive events. Sociology Compass, 16, e12972. 10.1111/soc4.12972 38895138 PMC11185416

[gbaf118-B7] Bildtgård T. , Öberg P. (2025). Uncoupling in the third age—the importance of the existential context for late-life divorce. Ageing and Society, 24, 100–122. 10.1017/S0144686X23000272

[gbaf118-B8] Booth A. , Amato P. (1991). Divorce and psychological stress. Journal of Health and Social Behavior, 32, 396–407. 10.2307/2137106 1765629

[gbaf118-B9] Brown S. L. , Lin I. F. (2012). The gray divorce revolution: Rising divorce among middle-aged and older adults, 1990–2010. The Journals of Gerontology, Series B: Psychological Sciences and Social Sciences, 67, 731–741. 10.1093/geronb/gbs089 23052366 PMC3478728

[gbaf118-B10] Brown S. L. , Lin I. F. (2022). The graying of divorce: A half century of change. The Journals of Gerontology, Series B: Psychological Sciences and Social Sciences, 77, 1710–1720. 10.1093/geronb/gbac057 35385579 PMC9434459

[gbaf118-B11] Büyükkeçeci Z. , Leopold T. (2021). Sibling influence on family formation: A study of social interaction effects on fertility, marriage, and divorce. Advances in Life Course Research, 47, 100359. 10.1016/j.alcr.2020.100359 36715429

[gbaf118-B12] Büyükkeçeci Z. , Leopold T. (2024). Parent-child relationships following grey divorce: Stronger ties with mothers, weaker ties with fathers. The Journals of Gerontology, Series B: Psychological Sciences and Social Sciences, 79, 1–9. 10.1093/geronb/gbae004 38280213

[gbaf118-B13] De Vuijst E. , Poortman A.-R., Das M., Van Gaalen R. (2017). Cross-sibling effects on divorce in the Netherlands. Advances in Life Course Research, 34, 1–9. 10.1016/j.alcr.2017.06.003

[gbaf118-B14] Dörfler-Bolt S. , Buchebner-Ferstl S., Kaindl M. (2022). Grey divorce in osterreich. Entwicklung, auslösende Mechanismen und Auswirkungen bei Personen mit höherem Scheidungsalter (p. 43). ÖIF Forschungsbericht. 10.25365/phaidra.319

[gbaf118-B15] Dupont E. , Van Pottelberge A., Van de Putte B., Lievens J., Caestecker F. (2020). Divorce in Turkish and Moroccan communities in Belgium. European Journal of Population, 36, 617–641. 10.1007/s10680-019-09545-w 32999638 PMC7492348

[gbaf118-B16] Dzamarija M. T. (Ed.). (2022). Eldre innvandrere i Norge. Demografi, boforhold, inntekt, formue og helse. Statistics Norway.

[gbaf118-B17] Engin C. , Hürman H., Harvey K. (2020). Marriage and family in Turkey: Trends and attitudes. In D. N. Farris, A. J. J. Bourque (Eds.), International handbook of population (Vol. 7, pp. 105–119). Springer International Publishing. 10.1007/978-3-030-35079-6_8

[gbaf118-B18] Festinger L. (1957). A theory of cognitive dissonance. Row and Peterson.

[gbaf118-B19] Flemmen A. B. (2008). Transnational marriages–Empirical complexities and theoretical challenges. An exploration of intersectionality. NORA—Nordic Journal of Feminist and Gender Research, 16, 114–129. 10.1080/08038740802140244

[gbaf118-B20] Fučík P. (2020). Trends in divorce acceptance and its correlates across European countries. Czech Sociological Review, 56, 863–895. 10.13060/csr.2020.053

[gbaf118-B21] Furtado D. , Marcén M., Sevilla A. (2013). Does culture affect divorce? Evidence from European immigrants in the United States. Demography, 50, 1013–1038. 10.1007/s13524-012-0180-2 23322379

[gbaf118-B22] Gloor S. , Gonin-Spahni S., Znoj H., Perrig-Chiello P. (2021). Repartnering and trajectories of life satisfaction after separation and divorce in middle and later life. Journal of Social and Personal Relationships, 38, 2205–2224. 10.1177/02654075211009594

[gbaf118-B23] Goldfarb M. R. , Trudel G. (2019). Marital quality and depression: A review. Marriage & Family Review, 55, 737–763. 10.1080/01494929.2019.1610136

[gbaf118-B24] Grundy E. , Van den Broek T., Keenan K. (2019). Number of children, partnership status, and later-life depression in Eastern and Western Europe. The Journals of Gerontology, Series B, 74, 353–363. 10.1093/geronb/gbx050 PMC6327656 28472400

[gbaf118-B25] Hogendoorn B. , Kalmijn M. (2024). Does ethnicity moderate the union dissolution penalty for women? A register-based analysis of changes in income components. European Journal of Population, 40, 29. 10.1007/s10680-024-09714-6 39388039 PMC11467139

[gbaf118-B26] Hogendoorn B. , Leopold T., Bol T. (2020). Divorce and diverging poverty rates: A risk-and-vulnerability approach. Journal of Marriage and Family, 82, 1089–1109. 10.1111/jomf.12629

[gbaf118-B27] Hu Y. , Metsä-Simola N., Malmberg S., Martikainen P. (2024). Trajectories of antidepressant use before and after union dissolution and re-partnering in later life: A prospective total population register-based cohort study. Journal of Epidemiology and Community Health, 78, 277–283. 10.1136/jech-2023-221529 38320855

[gbaf118-B28] Inglehart R. (2006). Mapping global values. Comparative Sociology, 5, 115–136. 10.1163/156913306778667401

[gbaf118-B29] Kalmijn M. (2007). Explaining cross-national differences in marriage, cohabitation, and divorce in Europe, 1990–2000. Population Studies, 61, 243–263. 10.1080/00324720701571806 17979001

[gbaf118-B30] Kalmijn M. , Uunk W. (2007). Regional value differences in Europe and the social consequences of divorce: A test of the stigmatization hypothesis. Social Science Research, 36, 447–468. 10.1016/j.ssresearch.2006.06.001

[gbaf118-B31] Kapelle N. , Monden C. (2024). Transitory or chronic? Gendered loneliness trajectories over widowhood and separation in older age. Journal of Health and Social Behavior, 65, 292–308. 10.1177/00221465231223719 38279812 PMC11144354

[gbaf118-B32] Kravdal Ø. , Hart R., Wörn J., Reme B. A. (2024). The influence of parental cancer on the mental health of children and young adults: Evidence from Norwegian register data on healthcare consultations. Demographic Research, 50, 763–796. 10.4054/DemRes.2024.50.27

[gbaf118-B33] Kravdal Ø. , Wörn J., Reme B.-A. (2023). Mental health benefits of cohabitation and marriage: A longitudinal analysis of Norwegian register data. Population Studies, 77, 91–110. 10.1080/00324728.2022.2063933 35502948

[gbaf118-B34] Krzyż E. Z. , Antunez Martinez O. F., Lin H. R. (2023). Uses of Andersen health services utilization framework to determine healthcare utilization for mental health among migrants—a scoping review. Frontiers in Public Health, 11, 1284784. 10.3389/fpubh.2023.1284784 38170142 PMC10761300

[gbaf118-B35] Kulu H. , Boyle P. (2010). Premarital cohabitation and divorce: Support for the “trial marriage” theory? Demographic Research, 23, 879–904. 10.4054/DemRes.2010.23.31

[gbaf118-B36] Laue J. , Diaz E., Eriksen L., Risør T. (2023). Migration health research in Norway: a scoping review. Scandinavian Journal of Public Health, 51, 381–390. 10.1177/14034948211032494 34609260 PMC10251465

[gbaf118-B37] Lin I. F. , Brown S. L. (2020). Consequences of later-life divorce and widowhood for adult well-being: A call for the convalescence model. Journal of Family Theory & Review, 12, 264–277. 10.1111/jftr.12366 PMC12439811 40964659

[gbaf118-B38] Lin I. F. , Brown S. L. (2021). The economic consequences of gray divorce for women and men. The Journals of Gerontology, Series B: Psychological Sciences and Social Sciences, 76, 2073–2085. 10.1093/geronb/gbaa157 32906147 PMC8599059

[gbaf118-B39] Lin I. F. , Brown S. L., Wright M. R., Hammersmith A. M. (2018). Antecedents of gray divorce: A life course perspective. The Journals of Gerontology, Series B: Psychological Sciences and Social Sciences, 73, 1022–1031. 10.1093/geronb/gbw164 27986850 PMC6093363

[gbaf118-B40] Lin I. F. , Brown S. L., Wright M. R., Hammersmith A. M. (2019). Depressive symptoms following later-life marital dissolution and subsequent repartnering. Journal of Health and Social Behavior, 60, 153–168. 10.1177/0022146519839683 30957562 PMC6565490

[gbaf118-B41] Marin I. B. , Fernández D., Ayuso-Mateos J. L., Leonardi M., ­Tobiasz-Adamczyk B., Koskinen S., Sanchez-Niubo A., Cristóbal-Narváez P. (2022). Healthy aging and late-life depression in Europe: Does migration matter? Frontiers in Medicine, 9. 10.3389/fmed.2022.866524 PMC9680089 36425106

[gbaf118-B42] Martens C. T. (2018). 6.7 Norway. In W. Apt (Ed.), Demographic change and migration (pp. 126–132). VDI/VDE.

[gbaf118-B43] McDermott R. , Fowler J. H., Christakis N. A. (2013). Breaking up is hard to do, unless everyone else is doing it too: Social network effects on divorce in a longitudinal sample. Social Forces, 92, 491–519. 10.1093/sf/sot096 PMC3990282 24748689

[gbaf118-B44] Prioux F. , Barbieri M. (2012). Recent demographic developments in France: Relatively low mortality at advanced ages. Population, 67, 493–550. 10.3917/popu.1204.0597 PMC3839870 24285939

[gbaf118-B45] Tønnessen M. (2020). Declined total fertility rate among immigrants and the role of newly arrived women in Norway. European Journal of Population, 36, 547–573. 10.1007/s10680-019-09541-0 32699540 PMC7363761

[gbaf118-B46] Tosi M. , Van den Broek T. (2020). Gray divorce and mental health in the United Kingdom. Social Science & Medicine, 256, 113030. 10.1016/j.socscimed.2020.113030 32450471

[gbaf118-B47] Uhlenberg P. , Myers M. A. P. (1981). Divorce and the elderly. The Gerontologist, 21, 276–282. 10.1093/geront/21.3.276 7239255

[gbaf118-B48] Van den Broek T. (2024). Trends in echtscheiding onder 50-plussers in Nederland. Geron, 26, 6. https://gerontijdschrift.nl/artikelen/trends-in-echtscheiding-onder-50-plussers-in-nederland/

[gbaf118-B49] Van den Broek T. , Fleischmann M. (2022). Gender differences in bodyweight change following COVID-19 lockdown measures in the Netherlands: A prospective longitudinal study. BMJ Open, 12, e054658. 10.1136/bmjopen-2021-054658 PMC10098263 35477883

[gbaf118-B50] Wang H. , Amato P. R. (2000). Predictors of divorce adjustment: Stressors, resources, and definitions. Journal of Marriage and Family, 62, 655–668. 10.1111/j.1741-3737.2000.00655.x

