# Supplementary material for: Gray divorce among migrants and non-migrants in Norway: trends and implications for mental healthcare use
Source: J Gerontol B Psychol Sci Soc Sci. 2025 Jun 27;80(8):gbaf118. doi: 10.1093/geronb/gbaf118 (PMC12343045; doi:10.1093/geronb/gbaf118)
Supplement: gbaf118_Supplementary_Data [file gbaf118_supplementary_data.docx]

***The Journals of Gerontology, Series B: Psychological Sciences and Social Sciences* Supplementary Material: van den Broek & Kravdal. Gray divorce among migrants and non-migrants in Norway: Trends and implications for mental healthcare use.**

**Supplementary Table 1. Country clusters.**

| Cluster name | Origin countries* |
| --- | --- |
| Other Nordic countries | Denmark; Faroe Islands; Finland; Greenland; Iceland; Sweden |
| Rest of Western Europe | Andorra; Austria; Belgium; Cyprus; France; Germany; Gibraltar; Great Britain; Greece; Ireland; Italy; Liechtenstein; Luxembourg; Malta; Monaco; Netherlands; Portugal; Spain; Switzerland |
| Eastern Europe | Albania; Belarus; Bosnia and Herzegovina; Bulgaria; Croatia; Czech Republic; Estonia; Georgia; Hungary; Kosovo; Latvia; Lithuania; Moldova; Montenegro; North Macedonia; Poland; Romania; Russia  Serbia; Slovakia; Slovenia; Ukraine |
| North Africa, Turkey and Middle East | Algeria; Bahrain; Egypt; Iran; Iraq; Israel; Jordan; Kuwait; Lebanon; Libya; Morocco; Palestine; Saudi Arabia; Syria; Tunisia; Turkey; Western Sahara; United Arab Emirates; Yemen |
| Asia (except Middle East) | Afghanistan; Armenia; Azerbaijan; Bangladesh; Bhutan; Brunei; Cambodia; China; East Timor; Hong Kong; India; Indonesia; Japan; Kazakhstan; Kyrgyzstan; Laos; Macau; Malaysia; Maldives;  Mongolia; Myanmar; Nepal; North Korea; Pakistan; Philippines; Singapore; South Korea; Sri Lanka; Taiwan; Tajikistan; Thailand;  Turkmenistan; Uzbekistan; Vietnam |

Note: * Only origin countries with observations in our dataset are listed here.


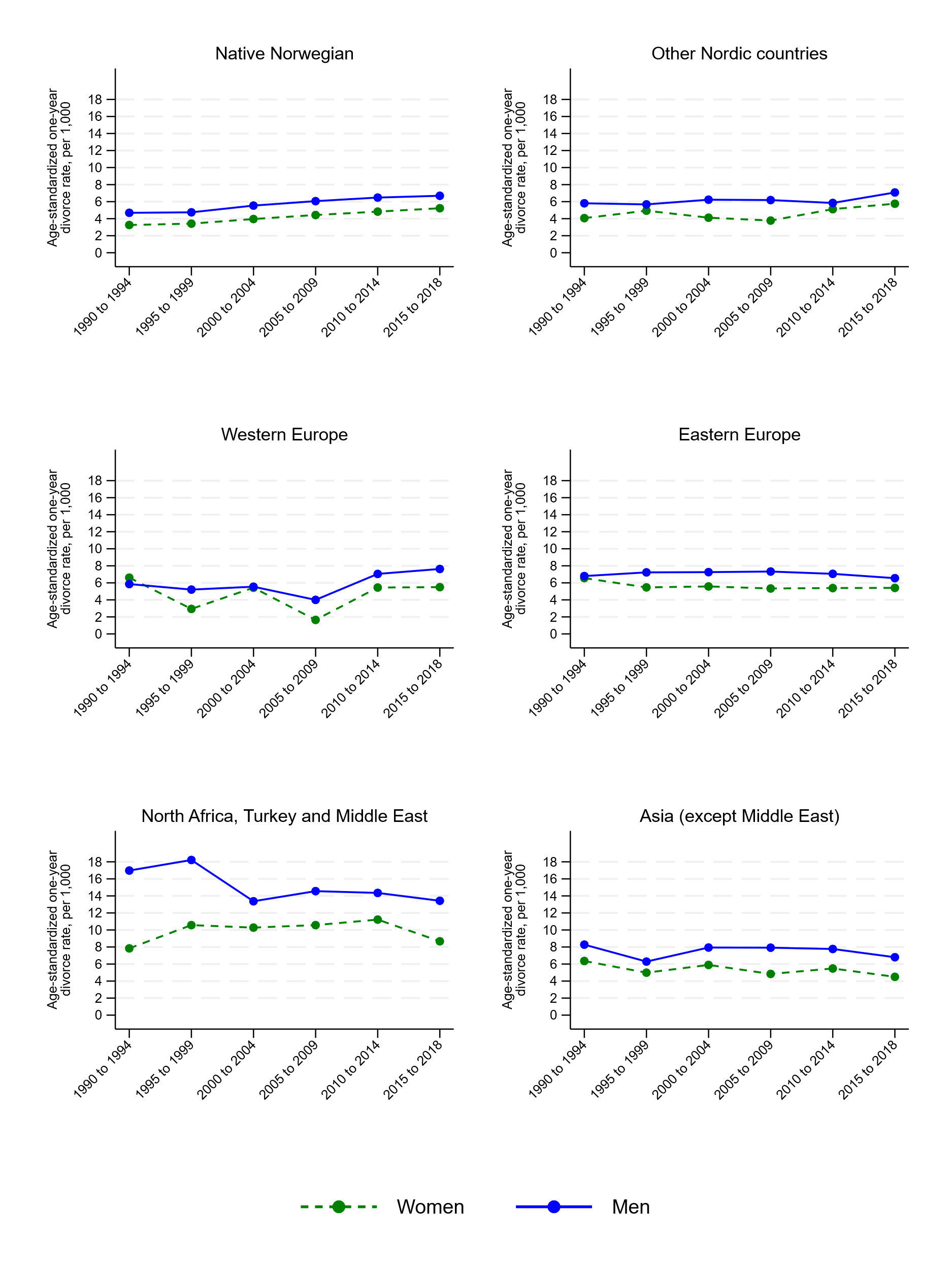


**Supplementary Figure 1. Trends in age-standardized one-year divorce rates by sex and origin group (For people in homogenous couples only).**

**Supplementary Table 2. Results of linear probability fixed effects regression analyses of mental healthcare use among women.**

|  | Native Norwegian | | Other Nordic countries | | Western Europe | | Eastern Europe | | North Africa, Turkey and Middle East | | Asia (except Middle East) | |
| --- | --- | --- | --- | --- | --- | --- | --- | --- | --- | --- | --- | --- |
|  | Coeff. | [95% C.I.] | Coeff. | [95% C.I.] | Coeff. | [95% C.I.] | Coeff. | [95% C.I.] | Coeff. | [95% C.I.] | Coeff. | [95% C.I.] |
| Time to / since divorce: |  |  |  |  |  |  |  |  |  |  |  |  |
| 5+ years pre-divorce | Ref. |  | Ref. |  | Ref. |  | Ref. |  | Ref. |  | Ref. |  |
| 3-4 years pre-divorce | 0.015** | [0.006;0.023] | 0.033 | [-0.018;0.085] | 0.032 | [-0.024;0.089] | 0.070** | [0.019;0.122] | -0.001 | [-0.068;0.066] | 0.050* | [0.007;0.094] |
| 1-2 years pre-divorce | 0.057*** | [0.047;0.066] | 0.034 | [-0.022;0.090] | 0.041 | [-0.022;0.104] | 0.080** | [0.029;0.131] | 0.046 | [-0.025;0.117] | 0.053* | [0.008;0.097] |
| Year of divorce | 0.177*** | [0.166;0.187] | 0.115*** | [0.047;0.183] | 0.110*** | [0.045;0.176] | 0.145*** | [0.088;0.203] | 0.120** | [0.037;0.203] | 0.127*** | [0.078;0.175] |
| 1-2 years post-divorce | 0.077*** | [0.067;0.087] | 0.029 | [-0.035;0.094] | 0.090** | [0.023;0.156] | 0.092** | [0.036;0.148] | 0.051 | [-0.030;0.133] | 0.068** | [0.022;0.115] |
| 3-4 years post-divorce | 0.026*** | [0.016;0.037] | 0.006 | [-0.062;0.075] | -0.009 | [-0.081;0.063] | 0.078** | [0.021;0.135] | 0.027 | [-0.061;0.114] | 0.059* | [0.008;0.111] |
| 5+ years post-divorce | -0.003 | [-0.014;0.008] | -0.023 | [-0.095;0.049] | -0.030 | [-0.107;0.047] | 0.034 | [-0.028;0.097] | -0.025 | [-0.117;0.066] | 0.022 | [-0.032;0.076] |
|  |  |  |  |  |  |  |  |  |  |  |  |  |
| Age: |  |  |  |  |  |  |  |  |  |  |  |  |
| 50 | Ref. |  | Ref. |  | Ref. |  | Ref. |  | Ref. |  | Ref. |  |
| 51 | 0.003** | [0.001;0.005] | 0.012 | [-0.000;0.024] | 0.006 | [-0.008;0.020] | 0.006 | [-0.004;0.016] | -0.002 | [-0.018;0.015] | 0.009* | [0.001;0.017] |
| 52 | 0.003** | [0.001;0.005] | 0.016* | [0.002;0.029] | 0.003 | [-0.013;0.018] | 0.005 | [-0.007;0.016] | -0.016 | [-0.035;0.002] | 0.013** | [0.004;0.022] |
| 53 | 0.005*** | [0.002;0.007] | 0.015* | [0.001;0.030] | 0.008 | [-0.008;0.024] | 0.017** | [0.004;0.029] | -0.028** | [-0.048;-0.008] | 0.011* | [0.001;0.020] |
| 54 | 0.008*** | [0.006;0.010] | 0.016* | [0.001;0.032] | 0.014 | [-0.004;0.031] | 0.019** | [0.006;0.032] | -0.037*** | [-0.059;-0.015] | 0.009 | [-0.001;0.020] |
| 55 | 0.010*** | [0.007;0.012] | 0.014 | [-0.002;0.030] | 0.013 | [-0.005;0.032] | 0.017* | [0.003;0.031] | -0.038** | [-0.062;-0.015] | 0.006 | [-0.004;0.017] |
| 56 | 0.009*** | [0.006;0.011] | 0.024** | [0.008;0.041] | 0.015 | [-0.005;0.034] | 0.016* | [0.001;0.031] | -0.049*** | [-0.073;-0.025] | 0.009 | [-0.002;0.020] |
| 57 | 0.010*** | [0.007;0.012] | 0.025** | [0.008;0.042] | 0.019 | [-0.000;0.039] | 0.026** | [0.010;0.041] | -0.066*** | [-0.092;-0.041] | 0.010 | [-0.002;0.021] |
| 58 | 0.011*** | [0.008;0.013] | 0.014 | [-0.004;0.031] | 0.027* | [0.006;0.047] | 0.014 | [-0.002;0.030] | -0.069*** | [-0.097;-0.042] | 0.013* | [0.000;0.025] |
| 59 | 0.011*** | [0.008;0.013] | 0.027** | [0.009;0.045] | 0.028** | [0.007;0.049] | 0.013 | [-0.004;0.031] | -0.076*** | [-0.105;-0.047] | 0.010 | [-0.003;0.023] |
| 60 | 0.009*** | [0.007;0.012] | 0.028** | [0.009;0.047] | 0.022* | [0.000;0.044] | 0.014 | [-0.005;0.032] | -0.092*** | [-0.123;-0.062] | 0.009 | [-0.004;0.022] |
| 61 | 0.011*** | [0.008;0.013] | 0.025* | [0.005;0.044] | 0.020 | [-0.002;0.043] | 0.006 | [-0.014;0.025] | -0.104*** | [-0.135;-0.073] | 0.006 | [-0.008;0.020] |
| 62 | 0.006*** | [0.004;0.009] | 0.015 | [-0.005;0.034] | 0.023 | [-0.001;0.046] | -0.001 | [-0.021;0.019] | -0.121*** | [-0.153;-0.089] | 0.005 | [-0.010;0.020] |
| 63 | -0.001 | [-0.004;0.002] | 0.009 | [-0.011;0.029] | 0.028* | [0.004;0.053] | -0.011 | [-0.033;0.010] | -0.128*** | [-0.162;-0.093] | 0.009 | [-0.007;0.025] |
| 64 | -0.004** | [-0.007;-0.002] | 0.012 | [-0.008;0.033] | 0.022 | [-0.002;0.046] | -0.030** | [-0.052;-0.008] | -0.134*** | [-0.171;-0.098] | 0.011 | [-0.005;0.027] |
| 65 | -0.006*** | [-0.008;-0.003] | 0.011 | [-0.009;0.032] | 0.010 | [-0.015;0.035] | -0.035** | [-0.058;-0.012] | -0.157*** | [-0.196;-0.118] | 0.001 | [-0.016;0.018] |
| 66 | -0.009*** | [-0.012;-0.006] | 0.006 | [-0.015;0.027] | 0.003 | [-0.022;0.028] | -0.025* | [-0.049;-0.000] | -0.170*** | [-0.209;-0.131] | 0.005 | [-0.013;0.023] |
| 67 | -0.011*** | [-0.014;-0.008] | -0.006 | [-0.027;0.015] | -0.001 | [-0.027;0.024] | -0.041** | [-0.068;-0.015] | -0.167*** | [-0.209;-0.125] | -0.002 | [-0.020;0.017] |
| *Table continued on next page* |  |  |  |  |  |  |  |  |  |  |  |  |
| *Continued from previous page* |  |  |  |  |  |  |  |  |  |  |  |  |
| 68 | -0.011*** | [-0.013;-0.008] | 0.004 | [-0.018;0.026] | -0.002 | [-0.028;0.023] | -0.041** | [-0.068;-0.013] | -0.167*** | [-0.212;-0.121] | 0.010 | [-0.009;0.030] |
| 69 | -0.007*** | [-0.010;-0.004] | 0.002 | [-0.020;0.024] | 0.004 | [-0.023;0.030] | -0.034* | [-0.062;-0.005] | -0.195*** | [-0.238;-0.152] | 0.005 | [-0.015;0.025] |
| 70 | -0.003 | [-0.006;0.000] | 0.007 | [-0.016;0.029] | -0.002 | [-0.029;0.024] | -0.029 | [-0.060;0.002] | -0.190*** | [-0.237;-0.142] | 0.016 | [-0.006;0.039] |
| 71 | 0.001 | [-0.002;0.005] | 0.007 | [-0.016;0.030] | 0.016 | [-0.012;0.043] | -0.039* | [-0.070;-0.008] | -0.173*** | [-0.224;-0.122] | 0.006 | [-0.017;0.029] |
| 72 | 0.007*** | [0.004;0.011] | 0.010 | [-0.013;0.033] | 0.016 | [-0.012;0.044] | -0.034* | [-0.066;-0.001] | -0.194*** | [-0.243;-0.145] | 0.047** | [0.018;0.075] |
| 73 | 0.015*** | [0.012;0.018] | 0.014 | [-0.010;0.038] | 0.036* | [0.007;0.065] | -0.016 | [-0.051;0.019] | -0.145*** | [-0.203;-0.087] | 0.026 | [-0.001;0.054] |
| 74 | 0.022*** | [0.019;0.025] | 0.027* | [0.002;0.052] | 0.044** | [0.014;0.074] | -0.019 | [-0.056;0.018] | -0.188*** | [-0.245;-0.130] | 0.022 | [-0.008;0.051] |
| 75 | 0.033*** | [0.030;0.037] | 0.026* | [0.000;0.052] | 0.053** | [0.021;0.084] | 0.002 | [-0.038;0.042] | -0.179*** | [-0.237;-0.120] | 0.017 | [-0.013;0.046] |
| 76 | 0.042*** | [0.039;0.046] | 0.030* | [0.003;0.057] | 0.058*** | [0.026;0.090] | 0.005 | [-0.035;0.046] | -0.193*** | [-0.258;-0.128] | 0.023 | [-0.011;0.056] |
| 77 | 0.053*** | [0.049;0.057] | 0.047** | [0.018;0.075] | 0.068*** | [0.035;0.102] | 0.028 | [-0.015;0.072] | -0.197*** | [-0.263;-0.131] | 0.048* | [0.011;0.086] |
| 78 | 0.068*** | [0.064;0.072] | 0.060*** | [0.030;0.090] | 0.098*** | [0.062;0.134] | 0.000 | [-0.044;0.043] | -0.175*** | [-0.256;-0.094] | 0.067*** | [0.027;0.107] |
| 79 | 0.081*** | [0.077;0.085] | 0.073*** | [0.042;0.105] | 0.120*** | [0.081;0.159] | 0.014 | [-0.034;0.062] | -0.165*** | [-0.250;-0.080] | 0.066** | [0.022;0.109] |
| 80 | 0.098*** | [0.093;0.102] | 0.071*** | [0.037;0.104] | 0.136*** | [0.095;0.177] | 0.054 | [-0.001;0.109] | -0.104* | [-0.203;-0.006] | 0.074** | [0.029;0.119] |

*Notes: Coeff.: Coefficient estimate; 95% C.I.: 95% confidence interval; * p < .05, ** p < .01, *** p < .001.*

**Supplementary Table 3. Results of linear probability fixed effects regression analyses of mental healthcare use among men.**

|  | Native Norwegian | | Other Nordic countries | | Western Europe | | Eastern Europe | | North Africa, Turkey and Middle East | | Asia (except Middle East) | |
| --- | --- | --- | --- | --- | --- | --- | --- | --- | --- | --- | --- | --- |
|  | Coeff. | [95% C.I.] | Coeff. | [95% C.I.] | Coeff. | [95% C.I.] | Coeff. | [95% C.I.] | Coeff. | [95% C.I.] | Coeff. | [95% C.I.] |
| Time to / since divorce: |  |  |  |  |  |  |  |  |  |  |  |  |
| 5+ years pre-divorce | Ref. |  | Ref. |  | Ref. |  | Ref. |  | Ref. |  | Ref. |  |
| 3-4 years pre-divorce | 0.016*** | [0.010;0.022] | -0.004 | [-0.041;0.033] | -0.008 | [-0.043;0.026] | 0.030 | [-0.021;0.080] | 0.026 | [-0.015;0.068] | -0.003 | [-0.043;0.037] |
| 1-2 years pre-divorce | 0.044*** | [0.038;0.051] | 0.067** | [0.024;0.109] | 0.032 | [-0.007;0.071] | 0.045 | [-0.011;0.101] | 0.044* | [0.000;0.088] | 0.011 | [-0.030;0.053] |
| Year of divorce | 0.121*** | [0.114;0.129] | 0.120*** | [0.074;0.166] | 0.119*** | [0.074;0.164] | 0.115*** | [0.056;0.174] | 0.084*** | [0.036;0.132] | 0.084*** | [0.038;0.129] |
| 1-2 years post-divorce | 0.055*** | [0.048;0.062] | 0.026 | [-0.019;0.071] | 0.043 | [-0.000;0.087] | 0.055 | [-0.006;0.116] | 0.039 | [-0.009;0.088] | 0.033 | [-0.010;0.076] |
| 3-4 years post-divorce | 0.024*** | [0.017;0.032] | 0.017 | [-0.032;0.067] | 0.014 | [-0.035;0.062] | 0.009 | [-0.057;0.075] | 0.009 | [-0.044;0.063] | 0.027 | [-0.021;0.076] |
| 5+ years post-divorce | 0.006 | [-0.002;0.014] | 0.001 | [-0.051;0.053] | -0.005 | [-0.058;0.048] | -0.026 | [-0.099;0.046] | -0.032 | [-0.090;0.027] | 0.020 | [-0.031;0.071] |
|  |  |  |  |  |  |  |  |  |  |  |  |  |
| Age: |  |  |  |  |  |  |  |  |  |  |  |  |
| 50 | Ref. |  | Ref. |  | Ref. |  | Ref. |  | Ref. |  | Ref. |  |
| 51 | 0.002** | [0.001;0.004] | 0.007 | [-0.004;0.018] | 0.004 | [-0.006;0.015] | 0.005 | [-0.002;0.012] | 0.002 | [-0.009;0.014] | -0.007 | [-0.015;0.002] |
| 52 | 0.003*** | [0.001;0.005] | 0.005 | [-0.007;0.017] | 0.004 | [-0.008;0.016] | 0.011** | [0.003;0.019] | -0.006 | [-0.020;0.007] | -0.002 | [-0.012;0.008] |
| 53 | 0.005*** | [0.003;0.007] | 0.012 | [-0.001;0.025] | 0.010 | [-0.002;0.023] | 0.010* | [0.002;0.019] | -0.006 | [-0.020;0.008] | -0.005 | [-0.015;0.005] |
| 54 | 0.007*** | [0.005;0.009] | 0.007 | [-0.006;0.021] | 0.011 | [-0.002;0.024] | 0.010* | [0.001;0.019] | -0.017* | [-0.032;-0.001] | 0.003 | [-0.008;0.013] |
| 55 | 0.008*** | [0.006;0.010] | 0.014 | [-0.000;0.028] | 0.010 | [-0.004;0.024] | 0.010* | [0.001;0.020] | -0.026** | [-0.042;-0.010] | 0.006 | [-0.005;0.018] |
| 56 | 0.010*** | [0.008;0.012] | 0.014 | [-0.000;0.029] | 0.016* | [0.002;0.031] | 0.018*** | [0.008;0.028] | -0.031*** | [-0.048;-0.013] | 0.010 | [-0.002;0.022] |
| 57 | 0.013*** | [0.011;0.015] | 0.009 | [-0.006;0.025] | 0.015* | [0.000;0.030] | 0.020*** | [0.009;0.031] | -0.036*** | [-0.054;-0.017] | 0.012 | [-0.001;0.025] |
| 58 | 0.015*** | [0.013;0.017] | 0.014 | [-0.002;0.030] | 0.024** | [0.008;0.040] | 0.018** | [0.006;0.030] | -0.042*** | [-0.062;-0.023] | 0.002 | [-0.011;0.015] |
| 59 | 0.015*** | [0.013;0.017] | 0.025** | [0.008;0.041] | 0.027** | [0.011;0.044] | 0.028*** | [0.015;0.041] | -0.053*** | [-0.074;-0.032] | 0.005 | [-0.008;0.018] |
| 60 | 0.016*** | [0.014;0.018] | 0.024** | [0.007;0.041] | 0.019* | [0.003;0.036] | 0.022** | [0.008;0.035] | -0.071*** | [-0.093;-0.049] | 0.002 | [-0.012;0.016] |
| 61 | 0.017*** | [0.015;0.019] | 0.025** | [0.008;0.042] | 0.022* | [0.005;0.040] | 0.013 | [-0.001;0.027] | -0.071*** | [-0.095;-0.048] | 0.005 | [-0.010;0.020] |
| 62 | 0.015*** | [0.013;0.018] | 0.024** | [0.007;0.042] | 0.022* | [0.005;0.040] | 0.008 | [-0.008;0.024] | -0.088*** | [-0.112;-0.064] | 0.001 | [-0.015;0.016] |
| 63 | 0.011*** | [0.009;0.013] | 0.018* | [0.000;0.036] | 0.014 | [-0.004;0.032] | 0.003 | [-0.013;0.020] | -0.103*** | [-0.128;-0.078] | -0.016* | [-0.032;-0.001] |
| 64 | 0.010*** | [0.008;0.012] | 0.014 | [-0.004;0.032] | 0.020* | [0.002;0.039] | -0.015 | [-0.033;0.002] | -0.120*** | [-0.147;-0.093] | -0.008 | [-0.025;0.008] |
| 65 | 0.010*** | [0.008;0.012] | 0.017 | [-0.002;0.035] | 0.014 | [-0.005;0.032] | -0.010 | [-0.029;0.009] | -0.124*** | [-0.153;-0.095] | -0.017 | [-0.033;0.000] |
| 66 | 0.008*** | [0.005;0.010] | 0.022* | [0.003;0.042] | 0.009 | [-0.010;0.028] | -0.022* | [-0.042;-0.002] | -0.140*** | [-0.169;-0.110] | -0.022* | [-0.039;-0.005] |
| 67 | 0.005*** | [0.003;0.008] | 0.014 | [-0.005;0.034] | 0.004 | [-0.015;0.023] | -0.029** | [-0.049;-0.009] | -0.149*** | [-0.179;-0.119] | -0.026** | [-0.044;-0.008] |
| *Table continued on next page* |  |  |  |  |  |  |  |  |  |  |  |  |
| *Continued from previous page* |  |  |  |  |  |  |  |  |  |  |  |  |
| 68 | 0.005*** | [0.002;0.007] | 0.010 | [-0.010;0.029] | -0.013 | [-0.032;0.007] | -0.022* | [-0.044;-0.001] | -0.162*** | [-0.193;-0.132] | -0.018 | [-0.037;0.001] |
| 69 | 0.007*** | [0.005;0.010] | 0.012 | [-0.008;0.032] | 0.000 | [-0.020;0.020] | -0.032** | [-0.055;-0.009] | -0.156*** | [-0.188;-0.124] | -0.017 | [-0.037;0.002] |
| 70 | 0.009*** | [0.007;0.012] | 0.019 | [-0.001;0.040] | -0.001 | [-0.021;0.020] | -0.019 | [-0.043;0.006] | -0.156*** | [-0.190;-0.123] | -0.015 | [-0.035;0.006] |
| 71 | 0.013*** | [0.010;0.015] | 0.027* | [0.005;0.048] | -0.005 | [-0.026;0.015] | -0.026* | [-0.051;-0.001] | -0.167*** | [-0.200;-0.134] | -0.026* | [-0.047;-0.005] |
| 72 | 0.017*** | [0.015;0.020] | 0.022* | [0.001;0.044] | 0.006 | [-0.015;0.027] | -0.042** | [-0.068;-0.017] | -0.170*** | [-0.205;-0.135] | -0.015 | [-0.036;0.007] |
| 73 | 0.024*** | [0.021;0.026] | 0.029* | [0.007;0.051] | 0.009 | [-0.013;0.030] | -0.031* | [-0.058;-0.005] | -0.168*** | [-0.205;-0.131] | -0.005 | [-0.029;0.018] |
| 74 | 0.031*** | [0.028;0.034] | 0.034** | [0.011;0.057] | 0.016 | [-0.008;0.039] | -0.026 | [-0.054;0.001] | -0.143*** | [-0.183;-0.103] | -0.005 | [-0.029;0.019] |
| 75 | 0.036*** | [0.033;0.039] | 0.033** | [0.009;0.057] | 0.027* | [0.003;0.052] | -0.013 | [-0.043;0.017] | -0.133*** | [-0.175;-0.092] | 0.017 | [-0.008;0.043] |
| 76 | 0.045*** | [0.042;0.048] | 0.060*** | [0.033;0.087] | 0.024 | [-0.002;0.049] | -0.013 | [-0.045;0.019] | -0.123*** | [-0.170;-0.075] | 0.011 | [-0.017;0.040] |
| 77 | 0.055*** | [0.052;0.058] | 0.057*** | [0.028;0.085] | 0.038** | [0.010;0.066] | 0.016 | [-0.019;0.052] | -0.162*** | [-0.207;-0.117] | 0.028 | [-0.001;0.057] |
| 78 | 0.067*** | [0.063;0.070] | 0.066*** | [0.037;0.095] | 0.033* | [0.005;0.060] | 0.018 | [-0.020;0.055] | -0.125*** | [-0.174;-0.075] | 0.017 | [-0.013;0.047] |
| 79 | 0.081*** | [0.077;0.084] | 0.069*** | [0.039;0.099] | 0.039* | [0.008;0.070] | 0.029 | [-0.009;0.066] | -0.124*** | [-0.174;-0.073] | 0.036* | [0.001;0.072] |
| 80 | 0.091*** | [0.087;0.094] | 0.083*** | [0.052;0.115] | 0.062*** | [0.028;0.095] | 0.062** | [0.018;0.106] | -0.106*** | [-0.165;-0.047] | 0.043* | [0.004;0.081] |

*Notes: Coeff.: Coefficient estimate; 95% C.I.: 95% confidence interval; * p < .05, ** p < .01, *** p < .001.*


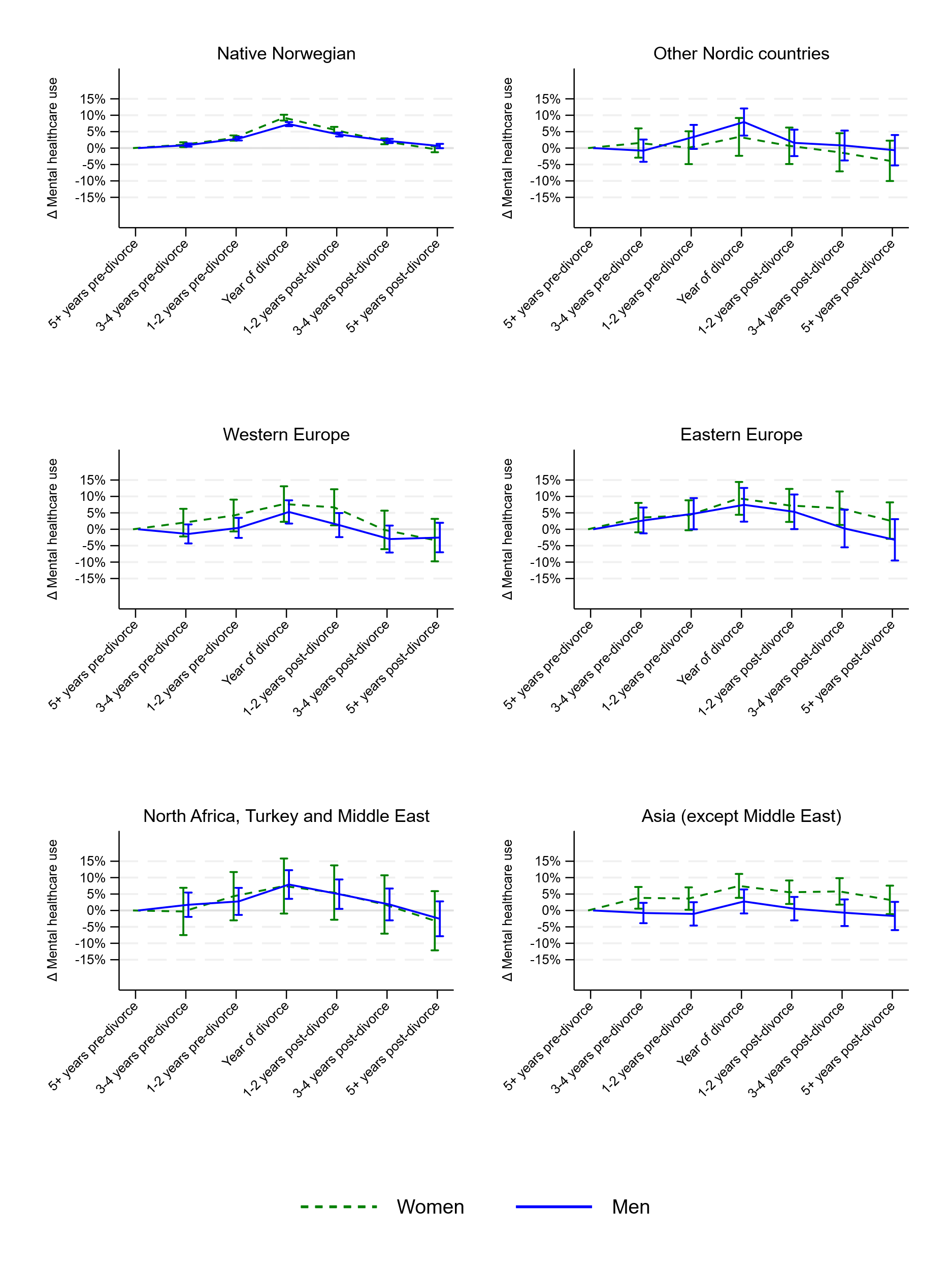


**Supplementary Figure 2. Estimated age-adjusted trajectories of mental healthcare use around gray divorce by sex and origin group, with 95% confidence intervals; Alternative outcome measure (healthcare use linked to diagnosis of mental health condition).**


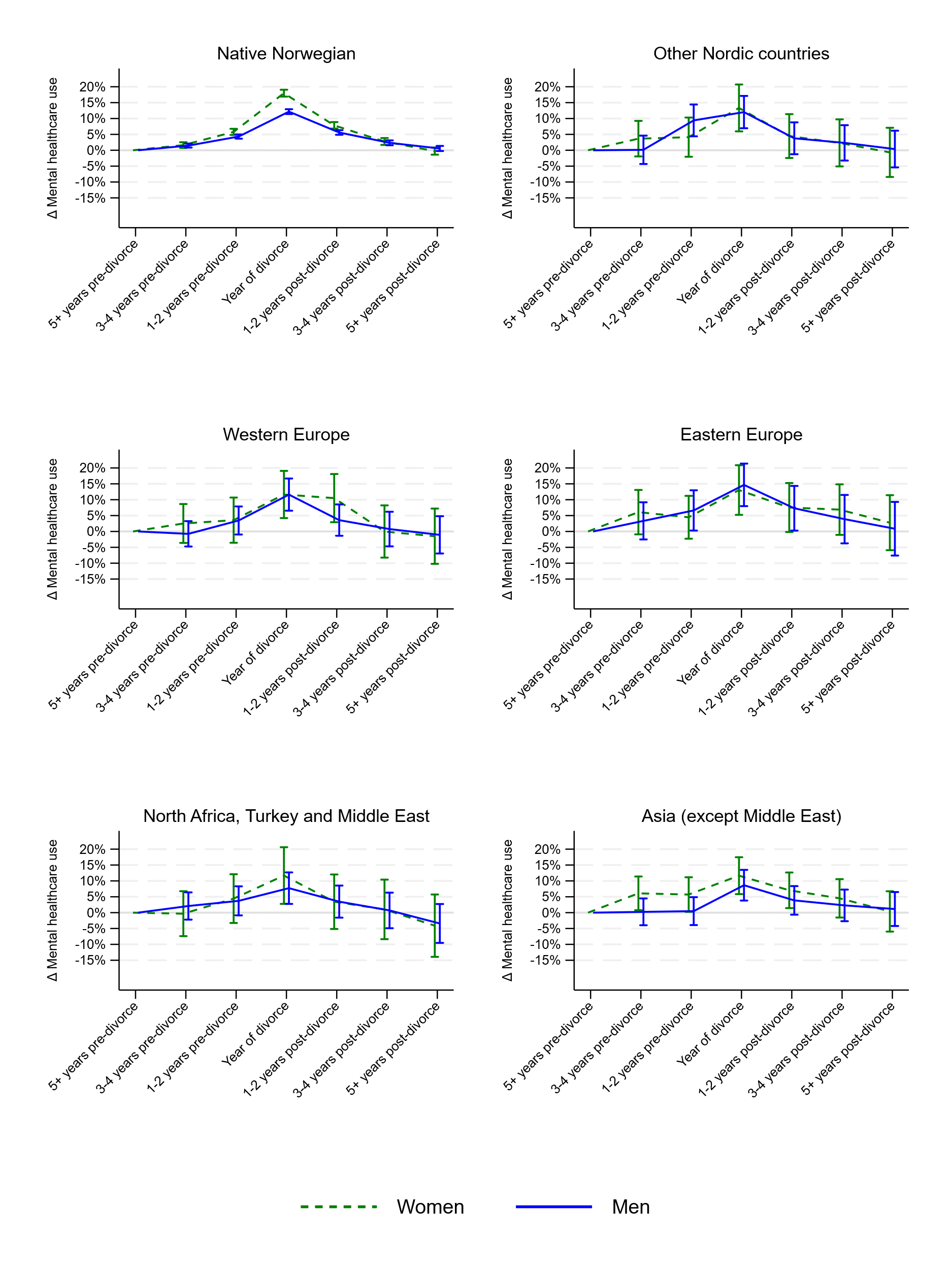


**Supplementary Figure 3. Estimated age-adjusted trajectories of mental healthcare use around gray divorce by sex and origin group, with 95% confidence intervals. Sample restricted to parents.**


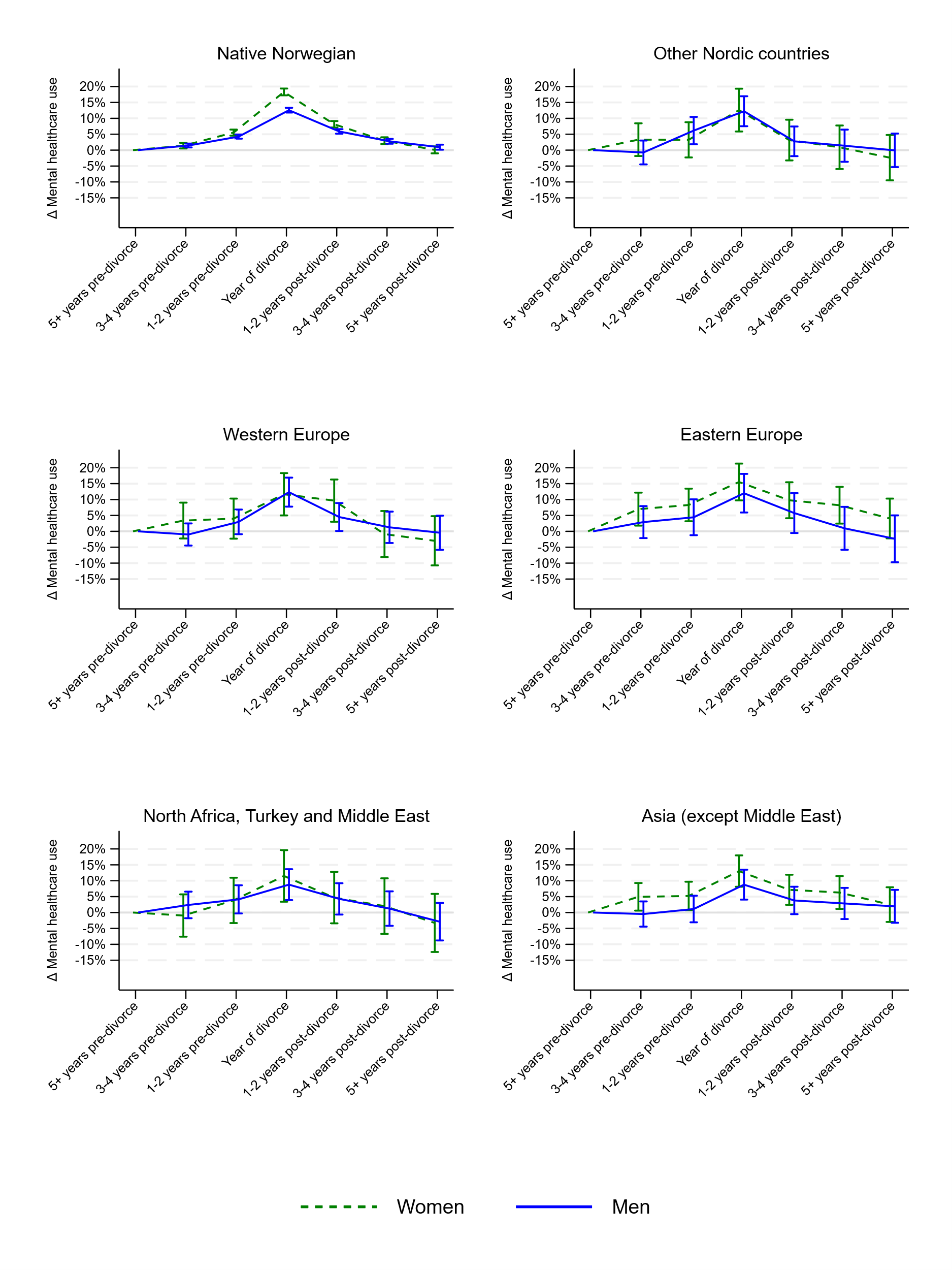


**Supplementary Figure 4. Estimated age-adjusted trajectories of mental healthcare use around gray divorce by sex and origin group, with 95% confidence intervals; Data are censored on the year of remarriage.**
